# Microbial Fermentation Enhances the Effect of Black Tea on Hyperlipidemia by Mediating Bile Acid Metabolism and Remodeling Intestinal Microbes

**DOI:** 10.3390/nu16070998

**Published:** 2024-03-28

**Authors:** Lingli Sun, Lianghua Wen, Qiuhua Li, Ruohong Chen, Shuai Wen, Xingfei Lai, Zhaoxiang Lai, Junxi Cao, Zhenbiao Zhang, Mengjiao Hao, Fanrong Cao, Shili Sun

**Affiliations:** 1 Tea Research Institute, Guangdong Provincial Key Laboratory of Tea Plant Resources Innovation and Utilization, Guangdong Academy of Agricultural Sciences, Guangzhou 510640, China; sunlingli@tea.gdaas.cn (L.S.); liqiuhua@tea.gdaas.cn (Q.L.); chenruohong@tea.gdaas.cn (R.C.); wenshuai@tea.gdaas.cn (S.W.); laixingfei@tea.gdaas.cn (X.L.); laizhaoxiang@tea.gdaas.cn (Z.L.); caojunxi@tea.gdaas.cn (J.C.); zhangzhenbiao@tea.gdaas.cn (Z.Z.); haomengjiao@gdaas.cn (M.H.); 2 College of Horticulture, South China Agricultural University, Guangzhou 510000, China; tea15779451015@163.com (L.W.); prcao@scau.edu.cn (F.C.)

**Keywords:** microbial fermented black tea, hyperlipidemia, bile acid, gut microbes

## Abstract

Black tea (BT), the most consumed tea worldwide, can alleviate hyperlipidemia which is a serious threat to human health. However, the quality of summer BT is poor. It was improved by microbial fermentation in a previous study, but whether it affects hypolipidemic activity is unknown. Therefore, we compared the hypolipidemic activity of BT and microbially fermented black tea (EFT). The results demonstrated that BT inhibited weight gain and improved lipid and total bile acid (TBA) levels, and microbial fermentation reinforced this activity. Mechanistically, both BT and EFT mediate bile acid circulation to relieve hyperlipidemia. In addition, BT and EFT improve dyslipidemia by modifying the gut microbiota. Specifically, the increase in *Lactobacillus johnsonii* by BT, and the increase in *Mucispirillum* and *Colidextribacter* by EFT may also be potential causes for alleviation of hyperlipidemia. In summary, we demonstrated that microbial fermentation strengthened the hypolipidemic activity of BT and increased the added value of BT.

## 1. Introduction

Economic growth has been accompanied by a dramatic rise in sedentary behavior and high-fat dietary intake, which has caused an increase in metabolic syndromes such as obesity, type 2 diabetes, hyperlipidemia, and non-alcoholic fatty liver disease [1,2]. One sort of dyslipidemia amongst them, hyperlipidemia, is characterized by weight increase and abnormal lipid measures [3,4]. Excessive blood lipid levels have been associated with increased blood viscosity, blood vessel blockage, and insufficient blood flow to some essential organs, and it is additionally connected to coronary heart disease, myocardial infarction, angina pectoris, cerebral thrombosis, stroke, and even a higher risk of COVID-19 and its associated complications [5,6]. Nearly half of all fatalities globally are caused by morbidity and mortality from hyperlipidemia and its consequences, which is a challenge for global public health [7]. Currently, statins and fibrates are the major therapeutic medications employed for managing hyperlipidemia; however, these medications come with a range of side effects, including the development of new-onset type 2 diabetes, the possibility of neurological and neurocognitive consequences, hepatitis, and renal toxicity [8]. As a consequence, developing novel therapies that are safe, effective, and natural has become an increasingly important research area in both academia and industry.

Dietary intervention has been demonstrated to be an efficient remedy for hyperlipidemia. Due to its multiple potential health advantages, including anti-tumor, anti-oxidant, anti-inflammatory, anti-viral, and anti-obesity properties, tea, a medicinal and edible crop, has attracted significant interest on a national and global level [9]. Black tea is the most popular tea in the world, not only for its flavor, but also for its potential health benefits, especially when it comes to fat reduction and weight loss. In fact, various research has reported that black tea alleviates obesity, hyperlipidemia, non-alcoholic fatty liver disease and cardiovascular disease by improving lipid levels [10,11,12]. Despite the beneficial effects of black tea in alleviating dyslipidemia, the heavy bitter taste, low aroma and poor quality of summer black tea have led to reluctance to consume it, resulting in a waste of resources. In our previous research, we proposed a microbial fermentation to increase the flavor of summer black tea, which reduces the waste of summer black tea to some extent [13], and microbial fermentation, which enhances the output of active chemicals in natural products or creates new active molecules [14,15]. Zhao et al. detected lovastatin, a clinical drug for hyperlipidemia, in microbially fermented pu-erh tea but not in unfermented pu-erh tea [16]. Therefore, we speculate that the employment of microbial fermentation not only improves the quality of black tea, but may also strengthen its hypolipidemic activity. In addition, previous relevant research has focused on the effect of black tea itself on lipid levels [10,11,12], and there are few reports on the adoption of other methods, especially microbial fermentation, to enhance the lipid-lowering activity of black tea.

The enterohepatic axis is believed to serve a significant role in the management of hyperlipidemia. The communication between the gut and liver depends primarily on the metabolites, with bile acids playing a crucial role [17]. Bile acids are catabolic products of cholesterol in the liver, and their dysregulation is associated with metabolic diseases, intestinal inflammation, and colorectal cancer [18]. Bile acids have even been suggested as treatment options for liver illness and obesity [19]. In addition, bile acids interact with gut microbes, which have been reported to be tightly connected to hyperlipidemia, and may be a new target for the therapeutic treatment of hyperlipidemia [20,21]. Wu et al. indicated that gut microbes are essential for berberine to ameliorate hyperlipidemia [22]. Accordingly, the homeostasis of bile acid metabolism and gut microbial communities is essential for ameliorating hyperlipidemia.

In the current study, we intended to assess whether microbial fermentation, while improving the black tea quality, enhances the efficacy of black tea in alleviating hyperlipidemia induced by a high-fat, high-fructose, and high-cholesterol diet (HFFCD). We evaluated the expression of key genes and proteins for bile acid metabolism using real-time qualitative polymerase chain reaction (RT-qPCR) and Western blot (WB), and assessed the composition of gut microbes using 16S rRNA sequencing. Our study will provide evidence that microbial fermented black tea mediates bile acid metabolism and remodels gut microbes to alleviate hyperlipidemia, and will contribute positively to the search for alternative strategies to ameliorate hyperlipidemia.

## 2. Materials and Methods

### 2.1. Preparation of Tea Extracts

The fresh tea was obtained from the Tea Research Institute of Guangdong Academy of Agricultural Sciences (Yingde, Guangdong Province, China). Following traditional processes, fresh tea was processed into BT [13]. The BT is then evenly sprayed with water to preserve its moisture level at 30–35%, stacked for 35 days to allow for natural microbial fermentation, and finally dried, which is processed into EFT [13]. The BT and EFT extracts were referred to the previous methods [23].

### 2.2. Analysis of the Main Components of Tea

The water extract content of tea was measured according to the Chinese standard (GB/T 8305-2013 [24]); the tea polyphenol content was identified by the Folin-Ciocalteu method (GB/T 8313-2018 [25]); the free amino acid was assessed by the ninhydrin colorimetry method (GB/T 8314-2013 [25]); and total soluble sugars were detected by the anthrone-sulfuric acid colorimetric method [26]; determination of flavonoids by the method of aluminum trichloride [26]; theaflavins, thearubigins, and theabrownins were measured with the People’s Republic of China Agricultural Industry Standard (NY/T 3675-2020 [27]).

### 2.3. High-Performance Liquid Chromatography (HPLC)

The following compounds were created as standard solutions in 50% methanol: gallic acid, gallocatechin, epi-gallocatechin, catechin, caffeine, epi-catechin, epi-gallocatechin-3-gallate, gallocatechin-3-gallate, epi-catechin-3-gallate, and catechin-3-gallate. Then, 95% methanol was used to extract the BT and EFT samples, and the filtrate was run through membranes with pore diameters of 0.45 μm. The Agilent 1260 HPLC system equipped with a Zorbax column (250 mm × 4.6 mm, 5 µm) was used to examine the tea samples. One milliliter per minute was used to elute the samples. Methanol (A) and 0.1% aqueous phosphoric acid (B) were used for elution, with 25% B applied from 0 to 19.5 min. The injection volume was 5 µL, and the sample was detected at 280 nm. At 30 °C, all procedures were completed.

### 2.4. Animal Experiments

Animal experiments were performed according to the Guidelines for Animal Care and Use of Tea Research Institute of Guangdong Academy of Agricultural Sciences and approved by the Animal Care and Use Committee of the Institute (Serial No. 2019003). Guangdong Zhiyuan Biomedical Technology Co., Ltd. (Guangzhou, China) supplied 50 male C57BL/6J mice that were between 7 and 8 weeks old. The animals were given unrestricted access to food and drink while being kept in a standard environment (25 ± 2 °C temperature, 55 ± 10% humidity, and a 12-h light/dark cycle beginning at 8:00 a.m.). Mice were randomly assigned to one of five treatment groups (*n* = 10) after 7 days of acclimatization: (1) Con group:normal maintenance chow diet; (2) Mod group: HFFCD; (3) Pos group:HFFCD with a daily intragastric gavage of 20 mg/kg simvastatin; (4) BT group:HFFCD with a daily intragastric gavage of BT extract; and (5) EFT group: HFFCD with a daily intragastric gavage of EFT extract. Diets were obtained from Xietong Bioengineering Co., Ltd. (Nanjing, China), and their compositions and energy densities are listed in Table S1. After 64 days of treatment, all mice were euthanized. Blood and tissue samples were collected, weighed, and rapidly frozen in liquid nitrogen and then stored at −80 °C.

### 2.5. Biochemical Assays

Blood and liver levels of total cholesterol TC, triglycerides (TG), low-density lipoprotein cholesterol (LDL-C), high-density lipoprotein cholesterol (HDL-C), and (TBA), as well as fecal levels of total bile acids, were measured using commercial kits (Nanjing Jiancheng Bioengineering Institute, Nanjing, China).

### 2.6. Histopathological Staining

The liver tissues were fixed in 10% formalin solution for 24 h and dehydrated in 75% ethanol for 24 h before being embedded in paraffin. And the blocks were divided into thin slices and stained with hematoxylin and eosin (H&E). The slides were analyzed with an Olympus microscope (Tokyo, Japan, 200× and 400×). Liver sections were evaluated histopathologically using a histologic scoring system for non-alcoholic fatty liver disease in reference to previous research [28].

### 2.7. Real-Time Quantitative Polymerase Chain Reaction (RT-qPCR)

Total RNA was extracted from liver and ileum using Total RNA Kit I (Omega Bio-Tek, Guangzhou, China), and cDNA was synthesized using a reverse transcription kit (TOYOBO Co., Ltd., Osaka, Japan). RT-qPCR was accomplished on an ABI7500 Real-Time System using SYBR Green PCR Master Mix (YEASEN, Shanghai, China). A three-step amplification program was used: pre-denaturation at 95 °C for 5 min, 40 cycles of 95 °C for 10 s, 60 °C for 20 s, and 72 °C for 20 s. All primer sequences were synthesized by Sangon Biotech Co., Ltd. (Shanghai, China) and are presented in Table S2. The relative gene expression levels were normalized to that of β-actin.

### 2.8. Western Blot

In total, 990 μL of radio immunoprecipitation assay (RIPA) mixed with 10 μL of phenylmethanesulfonyl fluoride (PMSF) was used to homogenize liver and ileum tissue samples (40 mg). The homogenates were placed on ice for 60 min before being centrifuged at 18,506 g for 20 min at 4 °C to remove debris. Bidicinchoninic acid (BCA, Thermo, Waltham, MA, USA) was utilized for determining the content of protein in the lysates. Equal amounts of each sample were combined with a quarter volume of loading buffer 4× and denatured for 5 min at 98 °C in a water bath. Using polyacrylamide gel electrophoresis (80–120 V), the proteins were separated and then transferred to a polyvinylidene fluoride (PVDF) membrane. After being blocked with 5% defatted milk solution for 2 h, the membranes were incubated overnight with primary antibodies specific for Farnesoid X-Activated Receptor (FXR, proteintech, Wuhan, China, diluted 1:1000), Cytochrome P450 27A1 (CYP27A1, proteintech, Wuhan, China, diluted 1:2000), Cholesterol 7alpha-hydroxylase (CYP7A1, Affinity, Liyang, China, diluted 1:1000), Apical sodium-dependent bile acid transporter (ASBT, Affinity, Liyang, China, diluted 1:500), β-actin (CST, Danvers, MA, USA, diluted 1:1000) and Glyceraldehyde-3-phosphate dehydrogenase (GAPDH, CST, Danvers, MA, USA, diluted 1:500) at 4 °C, followed by the secondary antibody at 37 ℃ for 50 min. After colorimetric detection and chemiluminescence imaging, the positive bands were quantified by densitometry using Image J software (open source image program for scientific images; http://imagej.net/ImageJ; accessed on 4 May 2023). The results were normalized to the density of GAPDH or β-actin bands.

### 2.9. Analysis of Gut Microbiota by 16S rRNA Sequencing

DNA from fresh feces was extracted by CTAB and labeled with specific barcodes and amplified with 16S specific primers. TruSeq^®^ DNA PCR-Free Sample Preparation Kit was used to construct the library. The amplified products were sequenced in NovaSeq6000. Sequencing data were filtered, spliced, and compared to obtain Effective Tags, which were generated using QIIME 2 (v2023.2) to generate Amplicon Sequence Variant (ASV) and analyzed for bioinformatics. Taxonomic information was obtained by performing species annotation analysis using the Mothur (v1.48) method with the SSUrRNA database from SILVA138.1. Alpha and beta diversity analyses were performed using R (v4.2.0) to determine species richness and diversity; For species analysis with substantial differences, linear discriminant analysis (LDA) utilizing linear discriminant effect size (LEfSe) was utilized. Raw data are stored in the Sequence Read Archive (BioProject ID: PRJNA1090409).

### 2.10. Statistical Analysis

The data are expressed as the mean ± standard error of the mean (SEM). For statistical analysis, GraphPad Prism 8.0 (GraphPad Software Inc., San Diego, CA, USA) was utilized. Duncan’s new multiple-range test (MRT) was used in conjunction with one-way ANOVA to evaluate the differences between the mean values for each group. It was deemed statistically significant when *p* < 0.05.

## 3. Results

### 3.1. Analysis of the Main Components of BT and EFT

**Figure 1:**
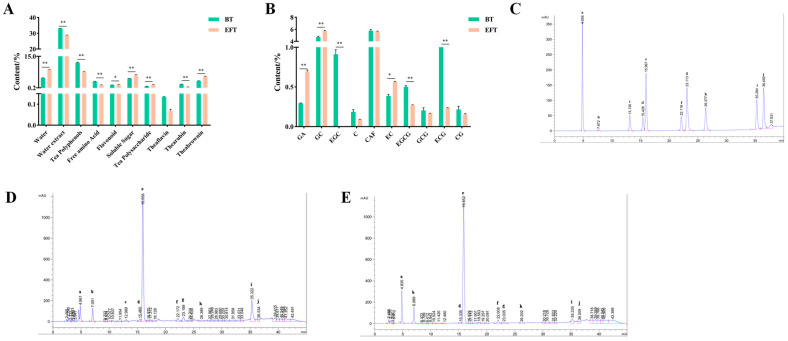
Analysis of the main components of BT and EFT. (**A**) The main compounds of BT and EFT. (**B**) The content of main phytochemicals in the BT and EFT. (**C**) The HPLC chromatograms of the standard samples. (**D**) The HPLC chromatograms of the BT. (**E**) The HPLC chromatograms of the EFT. (a, Gallic acid; b, Gallocatechin; c, Epi-gallocatechin; d, Catechin; e, Caffeine; f, Epi-catechin; g, Epi-gallocatechin-3-gallate; h, Gallocatechin-3-gallate; i, Epi-catechin-3-gallate; j, Catechin-3-gallate). Data are expressed as mean ± SEM; * *p* < 0.05 and ** *p* < 0.01.

During microbial fermentation, various bioactive components in the tea substrate undergo a series of biochemical reactions [29], so we analyzed the main components in BT and EFT. As shown in Figure 1A, the content of water extract, tea polyphenols, free amino acid, and thearubin in EFT was significantly lower, while the content of flavonoid, soluble sugar, tea polysaccharide, and theabrownin was significantly higher compared to BT. Also, the main phytochemical components of BT and EFT were identified and quantified by HPLC (Figure 1B–E). In comparison to BT, EFT revealed a large drop in epi-gallocatechin-3-gallate and epi-catechin-3-gallate content and a considerable rise in gallic acid, gallocatechin, and epi-catechin content. And there were no significant differences in the theaflavin, catechin, gallocatechin-3-gallate and catechin-3-gallate contents in BT and EFT. It is worth noting that we did not find EGC in EFT, but it was present in BT. Overall, these results indicate significant differences in the main components of BT and EFT after microbial fermentation.

### 3.2. Effect of BT and EFT on Body Weight and Liver

**Figure 2:**
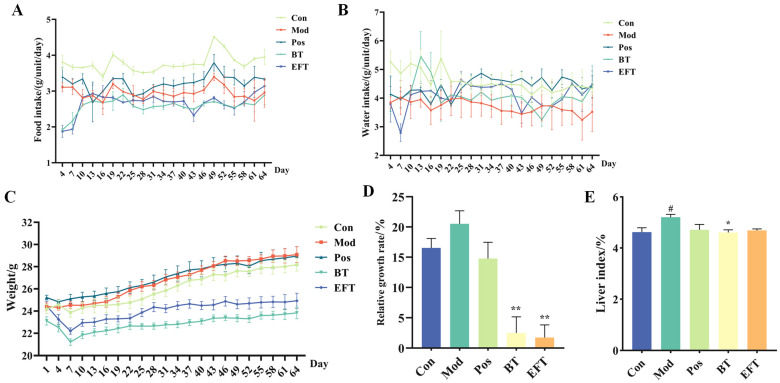
Effect of BT and EFT on body weight and liver weight. (**A**) Food intake. (**B**) Water intake. (**C**) Body weight. (**D**) Relative growth rate. (**E**) Liver index. Data are expressed as mean ± SEM; # *p* < 0.05 versus Con group; * *p* < 0.05 and ** *p* < 0.01 versus the Mod group.

C57BL/6J mice were fed with HFFCD and gavage with 800 mg/kg BW BT or EFT for 64 days to investigate the effect of BT and EFT against HFFCD-induced hyperlipidemia. During animal experiments, the amount of food and water consumed by the mice was steady in each group of mice, with the control group taking a greater amount of food (Figure 2A,B), which may be due to the better palatability of the maintenance diet compared to the HFFCD. Also, the body weight of the mice was recorded at three-day intervals, and the Con and Pos groups demonstrated a declining tendency compared to the Mod group, but it was not statistically significant, while the BT and EFT groups displayed a significant decrease in body weight (Figure 2C). Compared to BT, EFT enhanced the inhibition of weight gain as an effect (Figure 2D). Moreover, the Mod group had a higher liver index than the Con group; as expected, the Pos, BT, and EFT groups all had a lower liver index compared with the Mod group, but just the BT group was statistically significant (Figure 2E). The above findings indicated that microbial fermentation not only improved the flavor of black tea, but also enhanced the property of black tea to inhibit weight gain in hyperlipidemic mice.

### 3.3. Effect of BT and EFT on Lipid Level in Serum and Liver

**Figure 3:**
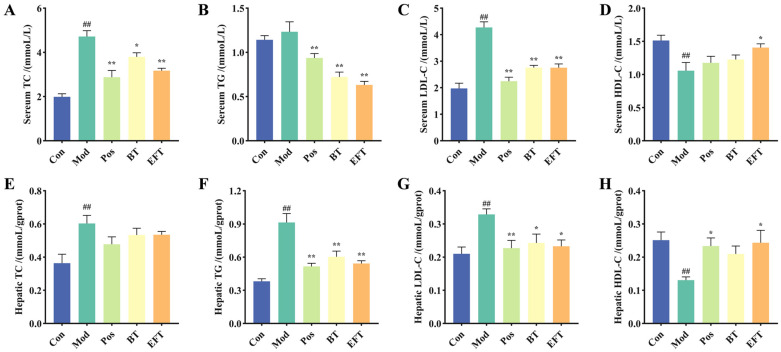
The lipid levels in liver and serum. (**A**) The TC in serum. (**B**) The TG in serum. (**C**) The LDL-C in serum. (**D**) The HDL-C in serum. (**E**) The TC in liver. (**F**) The TG in liver. (**G**) The LDL-C in liver. (**H**) The HDL-C in serum. Data are expressed as mean ± SEM; ## *p* < 0.01 versus Con group; * *p* < 0.05 and ** *p* < 0.01 versus the Mod group.

Hyperlipidemia is characterized by elevated levels of TC, TG, and LDL-C in the blood and decreased levels of HDL-C. We measured the levels of TC, TG, LDL-C and HDL-C in the serum, and compared with the Con group, the TC and LDL-C levels of the Mod group were significantly higher than those of the Con group, while the HDL-C levels were significantly lower than those of the Con group, while TG levels also showed a rising trend, but there was no significant difference (Figure 3A–D), which indicated that the hyperlipidemia mouse model was successfully established. In comparison to the Mod group, BT dramatically raised serum levels of HDL-C and decreased serum levels of TC, TG, and LDL-C (Figure 3A–D). Interestingly, compared with BT, EFT enhanced the improvement effect on TC, TG, LDL-C and HDL-C in serum. The liver serves as the metabolic center of the body and is the primary location of lipid synthesis [30], so we also measured the levels of TC, TG, LDL-C and HLD-C in the liver. The Mod group had higher levels of TC, TG, and LDL-C and lower levels of HDL-C compared with the Con group (Figure 3E–H). And this trend was reversed after oral administration of BT and EFT. Compared with the Mod group, BT reduced the levels of TC, TG, and LDL-C in the liver, and increased the HDL-C content. And compared with BT, EFT enhanced the inhibitory effects on TG and LDL-C, as well as the increase in HDL-C levels. In conclusion, our results indicated that BT and EFT were effective in attenuating HFFCD-induced abnormal lipid levels in serum and liver, and more importantly, illustrated that microbial fermentation not only improves the flavor of black tea, but also strengthens the bioactivity of black tea in ameliorating dyslipidemia.

### 3.4. Effect of BT and EFT on TBA Level in Serum, Liver, and Feces

**Figure 4:**
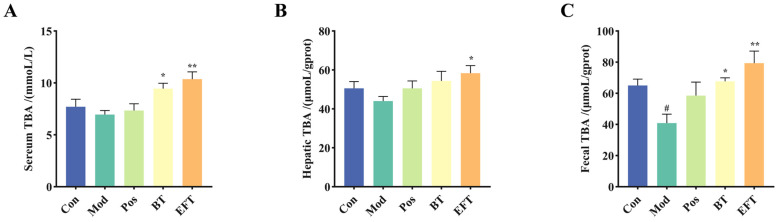
Analysis of TBA levels in serum, liver and feces. (**A**) Serum. (**B**) Liver. (**C**) Feces. Data are expressed as mean ± SEM; # *p* < 0.05 versus Con group; * *p* < 0.05 and ** *p* < 0.01 versus the Mod group.

A key element of hyperlipidemia is elevated cholesterol levels, and cholesterol is a biosynthetic precursor of bile acids, and bile acid biosynthesis is crucial to the control of systemic cholesterol homeostasis [31]. Therefore, we detected TBA levels in serum, liver and feces. The Mod group showed a lowering trend in TBA levels in blood, liver, and feces when compared to the Con group; however, there was a meaningful difference only in feces. (Figure 4). However, BT and EFT significantly increased TBA levels in liver, serum and feces compared to the Mod group. In addition, EFT enhanced TBA synthesis compared to BT, as we observed higher levels of TBA in liver, serum and feces in the EFT group. Taken together, these results provide important insights that black tea can promote bile acid biosynthesis and microbial fermentation enhances this property of black tea.

### 3.5. Analysis of Liver Histopathology

**Figure 5:**
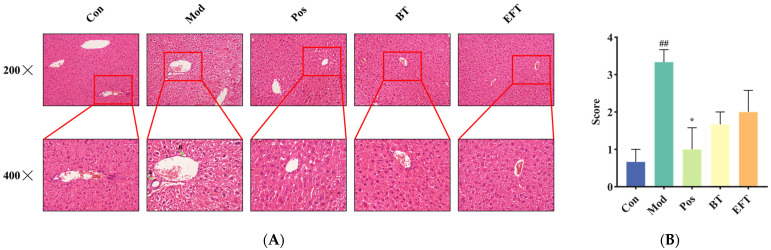
H&E staining of liver. (**A**) Representative liver section. (**B**) Histopathology score. Data are expressed as mean ± SEM; ## *p* < 0.01 versus Con group; * *p* < 0.05 versus the Mod group.

As mentioned above, HFFCD-induced hyperlipidemia leads to hepatic lipid accumulation. To understand the effects of BT and EFT on the liver structure of HFFCD-induced hyperlipidemic mice, we performed H&E staining of liver sections and scored the liver sections histopathologically with reference to previous studies [28]. From Figure 5, the liver structures in the Con group were neatly arranged and tightly packed, whereas the H&E staining in the Mod group showed infiltration of inflammatory cells and numerous balloon-like changes (as indicated by the green arrows in Figure 5A). However, these pathologic changes improved significantly after simvastatin, BT, and EFT interventions. Our findings indicate that BT and EFT supplementation are effective in ameliorating liver tissue lesions accompanying hyperlipidemia.

### 3.6. Effect of BT and EFT on Expression of Genes and Proteins in Bile Acid Metabolism Signaling Pathway

**Figure 6:**
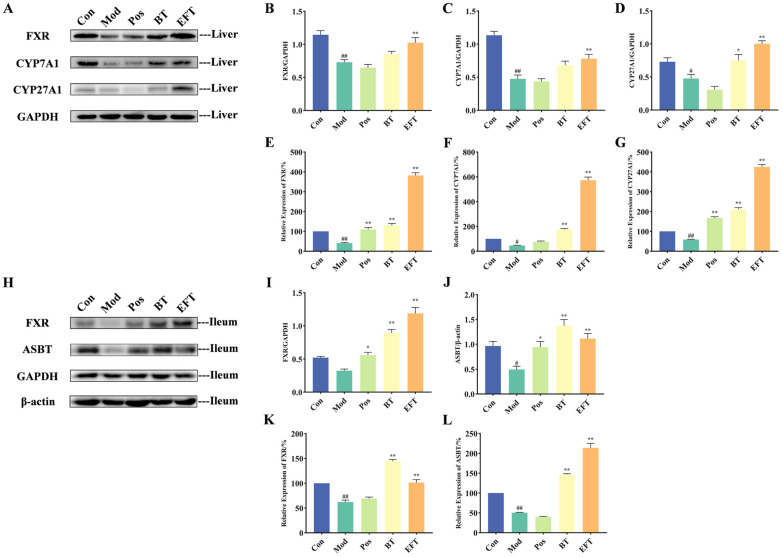
Effect of BT and EFT on expression of genes and proteins in bile acid metabolism signaling pathway. (**A**–**D**) Protein expression of hepatic FXR, CYP7A1 and CYP27A1. (**E–G**) Gene expression of hepatic FXR, CYP7A1 and CYP27A1. (**H**–**J**) Protein expression of ileac FXR and ASBT. (**K**,**L**) Gene expression of ileac FXR and ASBT. Data are expressed as mean ± SEM; # *p* < 0.05 and ## *p* < 0.01 versus Con group; * *p* < 0.05 and ** *p* < 0.01 versus the Mod group.

As demonstrated in the above results, BT and EFT interventions enhanced TBA production in HFFCD-induced hyperlipidemic mice, and thus we examined genes and protein expression of key targets in the bile acid metabolite signaling pathway. The expression of bile acid synthesis-related proteins, including FXR, CYP7A1, and CYP27A1, was significantly reduced in the liver of the Mod group compared with the Con group (Figure 6A–D). Notably, simvastatin did not appear to interfere with protein expression in bile acid metabolism in HFFCD-induced hyperlipidemic mice. However, supplementation with both BT and EFT promoted the expression of these proteins, with a significant increase in protein expression of FXR, CYP7A1, and CYP27A1 in the EFT group compared to the Mod group, but the effect of BT treatment was remarkable only for CYP27A1. With respect to gene expression of FXR, CYP7A1 and CYP27A1, the overall trend was similar to that of the corresponding protein expression. Briefly, the relative expression of these three genes was significantly downregulated in the Mod group, and the trend was significantly reversed by the BT and EFT interventions (Figure 6E–G). Interestingly, both at the protein level and at the gene level, EFT strengthened the expression of FXR, CYP7A1 and CYP27A1 in the liver compared to BT.

After bile acids converted from cholesterol are passed through the intestines, about 95% are reabsorbed (especially at the end of the ileum) and returned to the liver through the portal vein to maintain the bile acid cycle [32,33]. We examined the expression of proteins and genes of FXR and ASBT, which regulate bile acid reuptake. The protein and gene expressions of FXR and ASBT were downregulated in the Mod group compared with the Con group, which indicated abnormal bile acid reabsorption in HFFCD-induced hyperlipidemic mice (Figure 6H–L). However, simvastatin, BT, and EFT interventions reversed this trend and alleviated the abnormal bile acid reabsorption. Taken together, these results indicate that black tea contributes to the conversion of cholesterol into bile acids and that microbial fermentation improves the quality of black tea while also enhancing the property of black tea in promoting bile acid synthesis.

### 3.7. Effects of BT and EFT on the Gut Microbiota

The second largest genome in humans can be identified in the gut microbes [21], and increasing evidence suggests that it is tightly linked to host metabolism. We carried out 16S rRNA analyses of mice feces to obtain insight into how BT and EFT affected the gut microbial community in HFFCD-induced hyperlipidemic mice.

**Figure 7:**
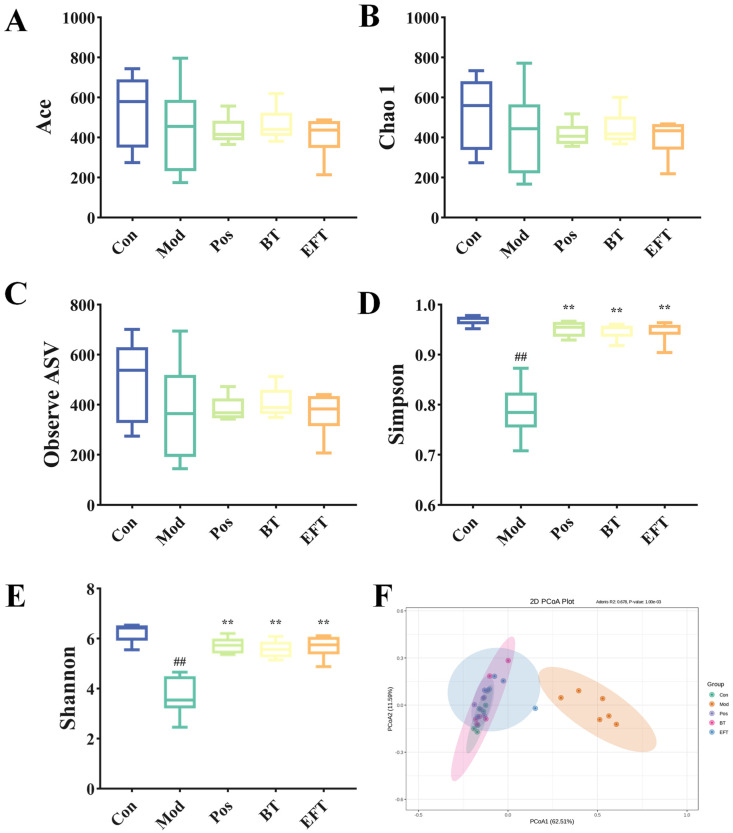
Effects of BT and EFT on gut microbial diversity. (**A**) Ace index; (**B**) Chao 1 index; (**C**) Observe ASV; (**D**) Simpson index; (**E**) Shannon index; (**F**) Principal coordinates analysis (PCoA) based on ASV level. Data are expressed as mean ± SEM; ## *p* < 0.01 versus Con group; ** *p* < 0.01 versus the Mod group.

The Q20 of all samples was greater than 97% and the Q30 was greater than 93%, which indicated that the 16S rRNA sequencing was of excellent quality (Table S3). And the Rarefaction curve indicated that the gut microbial richness detected in each group was saturated. The Abundance-based coverage estimator (ACE) index, Chao 1 index, and observed ASV were reduced in the Mod group compared to the Con group and were not relieved by supplementation with simvastatin, BT, and EFT (Figure 7A–C). However, downward revisions to the Simpson and Shannon indices were reversed (Figure 7D,E). These results suggest that BT and EFT do not improve the microbial community by restoring the number of gut microbial species, but rather by influencing gut microbial species composition and distribution. Further principal coordinates analysis (PCoA) showed that the microbial communities in the Mod group differed significantly from those in the Con, Pos, BT and EFT groups (Figure 7F).

**Figure 8:**
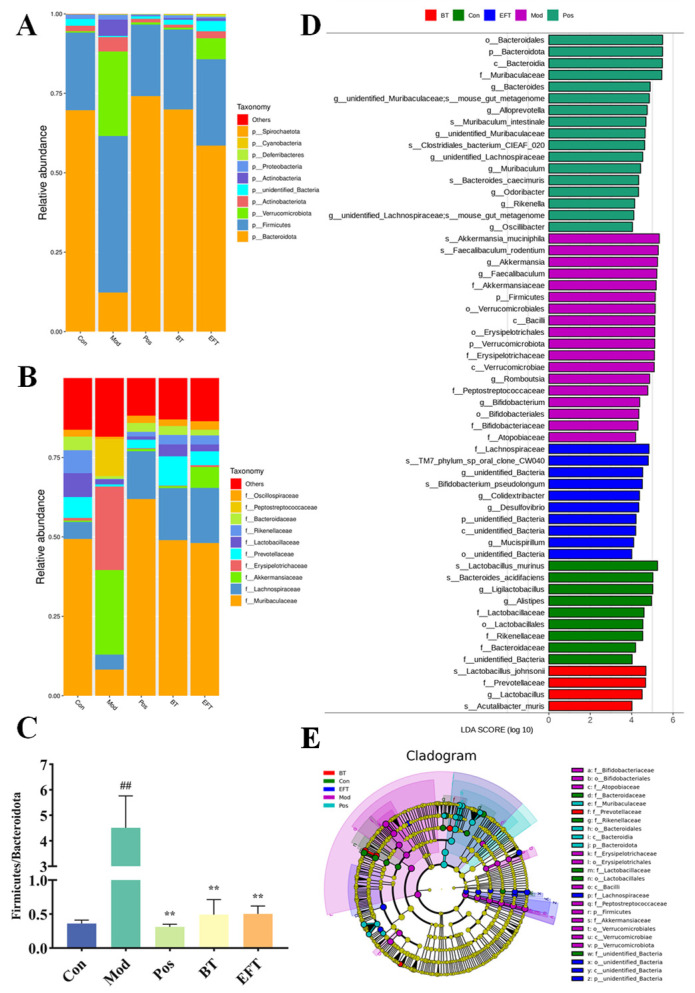
Effect of BT and EFT on gut microorganisms. (**A**) Microbial community composition at phylum level; (**B**) Microbial community composition at family level; (**C**) Ratio of Firmicutes to Bacteroidetes; (**D**) The linear discriminant analysis effect size (LefSe) of LDA score distribution histogram; (**E**) LEfSe analysis of an evolutionary branching diagram. Data are expressed as mean ± SEM; ## *p* < 0.01 versus Con group; ** *p* < 0.01 versus the Mod group.

At the phylum level, elevated Firmicutes, Verrucomicrobiota, Actinobacteriota, and Actinobacteria were significantly downregulated by BT and EFT, and Bacteroidota abundance in hyperlipidemic mice was upregulated by BT and EFT (Figure 8A). The ratio of Firmicutes to Bacteroidetes (F/B) has been reported to be strongly associated with metabolic syndrome, especially obesity [34]. The F/B ratio was significantly higher in the Mod group than in the Con group and was significantly downregulated after the BT and EFT interventions (Figure 8C). At the family level, *Muribaculaceae*, *Rikenellaceae*, and *Bacteroidaceae* abundance decreased in the Mod group, while *Akkermansiaceae*, *Erysipelotrichaceae*, and *Peptostreptococcaceae* abundance increased, and these shifts were reversed by BT and EFT (Figure 8B). To understand the key microorganisms in each group, we performed LefSe analysis of gut microorganisms in five groups. To understand the key microorganisms in each group, we performed LefSe analysis of gut microorganisms in five groups. We identified 58 richly differentiated taxa, including 20 genera. Among them, the predominant taxa in the Con group were *Alistipes* and *Ligilactobacillus*, whereas in the Mod group were *Akkermansia*, *Faecalibaculum*, *Romboutsia*, and *Bifidobacterium*. In addition, the main microorganism in the BT group was *Lactobacillus*, and *Colidextribacter*, *Desulfovibrio* and *Mucispirillum* prevailed in the EFT group (Figure 8D,E). These variations in microorganisms may suggest a potential mechanism for the development of hyperlipidemia. In short, our study demonstrates that BT and EFT may ameliorate hyperlipidemia by restoring the gut microbial community.

## 4. Discussion

Black tea is the most consumed tea in the world and also contributes to fat reduction and weight loss. Liao et al. compared the effects of Keemun black tea and Dianhong black tea on fatty liver and found that both of them could affect the expression of genes related to lipid metabolism [11]. Liu et al. also showed that black tea mediates the inhibition of high-fat diet-induced weight gain and lipid accumulation by intestinal microorganisms [12]. Microbial fermentation is a common method for food flavor improvement and innovation, and has also been reported to produce new active substances. However, previous studies have focused only on the effects of black tea supplementation on hyperlipidemia, and nothing is known about whether microbial fermentation alters the hypolipidemic activity of black tea. In this study, we evaluated the effect of microbial fermentation on the hypolipidemic activity of black tea and found that microbial fermentation significantly reinforced the hypolipidemic efficacy of black tea. Mechanistically, microbial fermentation enhanced the modulatory effects of black tea on the bile acid surrogate pathway and gut microbes.

The essence of microbial fermentation is the conversion of substances with the participation of microbial activity [35]. It is this that leads to differences in the biological activity of fermentation substrates. We observed a decrease in polyphenols, theaflavin, and thearubigins in BT, which could be caused by their oxidative polymerization to theabrownine [36], as we also observed a dramatic increase in theabrownine (Figure 1). Theabrownine has been reported to remodel the gut microbial community, inhibit lipogenesis, and improve hyperlipidemia [36]. In addition, tea polysaccharides as an active substance with potential health benefits have been observed to increase in EFT. A recent report claimed that tea polysaccharides reduce obesity and improve serum TC, TG, LDL-C, and HDL-C abnormalities by modulating gut microbes and their associated short-chain fatty acid and amino acid metabolism [37]. In accordance with other reports, the gallic acid content of tea increases after fermentation, and gallic acid alleviates hypertriglyceridemia and fat accumulation by modulating glycolytic and lipolytic pathways in perirenal adipose tissue [38,39]. Furthermore, we observed an increase in the levels of gallocatechin and epi-catechin in the EFT, while gallocatechin and epi-catechin also seemed to have favorable effects in improving lipid metabolism. gallocatechin positively contributes to the activation of Adenosine 5′-monophosphate-activated protein kinase (AMPK), which is closely related to lipolysis [40,41]. After 12 consecutive weeks of epi-catechin supplementation in high-fat, high-cholesterol-induced hyperlipidemic rats, TC, LDL-C, and TG levels were significantly reduced, and hepatic fat accumulation was alleviated, along with an increase in HDL-C levels, and a decrease in lipid peroxidation and pro-inflammatory cytokine production [42]. It may be that changes in the content of these actives are responsible for the stronger hypolipidemic activity of EFT than of BT, but further research is needed to determine which of these actives play a role.

The primary route for cholesterol metabolism in the liver is the transformation of cholesterol to bile acids [43]. And there are two pathways for the generation of bile acids: the classic pathway, which is controlled by CYP7A1, and the alternative pathway, which is mediated by CYP27A1. The nuclear receptor FXR controls the activity of these two essential enzymes [3,44]. HDL carries cholesterol from peripheral tissues to the liver, where it is transformed into bile acids, and the biliary system then transports the created bile acids to the bile ducts, where they are ultimately expelled into the intestines [44,45]. In order to preserve the bile acid cycle progressing, the majority of the bile acids are reabsorbed by the ileal epithelium through ASBT-mediated processes and then sent back to the liver via the portal vein [44]. Dietary intervention in bile acid metabolism has long been recognized as an effective strategy for alleviating hyperlipidemia. According to Duan et al., whole-grain oat-derived flavonoids were helpful in accelerating the transformation of cholesterol into bile acids, which reduced blood lipid levels [3]. In our research, we found that both BT and EFT promoted the expression of FXR, CYP7A1, and CYP27A1 in the liver, suggesting that the BT and EFT interventions effectively enhanced the conversion of cholesterol to bile acids; on the other hand, BT and EFT also increased the expression of ASBT and FXR in the ileum, further suggesting that BT and EFT facilitated the bile acid cycle. It has also been demonstrated that a chronic high-fat diet disrupts the intestinal barrier function, increases the body’s susceptibility to harmful intestinal substances, and leads to a systemic low-inflammatory state [46]. In addition, reduced bile acids can further exacerbate intestinal barrier dysfunction [47]. However, intestinal FXR activation contributes positively to inflammatory suppression and intestinal barrier protection [48]. Compared to the Mod group, we observed elevated bile acid levels and activated FXR expression in the BT and EFT groups, which revealed a protective effect of BT and EFT on the intestinal barrier. It is noteworthy that the effect of EFT was superior to that of BT, both in promoting hepatic bile acid synthesis and intestinal FXR activation, which indicates that hypolipidemic activity and intestinal protection of black tea are enhanced by microbial fermentation.

There is a strong association between Firmicutes and dietary energy intake as well as obesity, and a higher abundance of Firmicutes has been previously reported in populations with higher levels of obesity [49]. We observed that BT and EFT dramatically reduced the abundance of Firmicutes. In addition, Jiang et al. reported that hyperlipidemia led to an extreme reduction in Bacteroidetes [33], which is consistent with our current results, and gratefully BT with EFT reversed this alteration. Bacteroidetes and Firmicutes which have a critical role in energy metabolism in the gut, and the F/B ratio, which is closely related to the metabolic capacity of the microbial community and inflammation, are important indicators of gut ecological dysregulation associated with the metabolic syndrome [50,51]. A significant increase in the ratio of F/B in HFFCD-induced hyperlipidemic mice was also observed in the present research, whereas supplementation with BT and EFT resulted in its essentially return to normal levels. Similar effects are consistent with those reported by Duan et al. who also found a downregulation of both Firmicutes and F/B ratios and an increase in Bacteroidetes abundance after supplementing hyperlipidemic mice with flavonoids [3]. The available evidence suggests that the improvement of hyperlipidemia by BT and EFT may be the result of targeting these microorganisms.

*Lactobacillus* can alleviate lipid accumulation associated with a high-fat diet, and *Lactobacillus johnsonii* in particular is thought to adhere to intestinal epithelial cells and have serum cholesterol-lowering properties [52,53]. Zhu et al. also demonstrated that bile salt hydrolase from *Lactobacillus johnsonii* could mediate the FXR pathway to exert a hypocholesterolemic effect [54]. *Lactobacillus johnsonii* is a major microbiota in the BT group, perhaps this is a potential mechanism for the hypolipidemic efficacy of BT supplementation. An increasing number of research studies has suggested that a potential mechanism for dietary intervention to alleviate hyperlipidemia is the mediated production of short-chain fatty acids [55]. In the present study, hyperlipidemic mice supplemented with EFT were significantly enriched for *Mucispirillum*, a microorganism that typically produces short-chain fatty acids. Chlorogenic acid supplementation was also previously reported to increase *Mucispirillum* abundance and prevent obesity and related endotoxemia [56]. Ma et al. also observed an increase in *Mucispirillum* abundance as well as a significant elevation in short-chain fatty acid content in their study of *Lyciumbarbarum* Polysaccharide for improvement of type 2 diabetes, which further confirms that *Mucispirillum* may be a key strain in EFT for improvement of hyperlipidemia [57]. Chronic inflammation induces hyperlipidemia [58], and *Colidextribacter*, an anti-inflammatory probiotic [59] which is one of the major microorganisms in the EFT group, may alleviate hyperlipidemia by reducing inflammation in the body and enhancing the intestinal barrier function. Zhao et al. supplemented Sodium Alginate in rats fed a high-fat diet, increased the abundance of *Colidextribacter*, and effectively alleviated non-alcoholic fatty liver and serum lipid levels [60]. In addition, *Faecalibaculum* was positively correlated with serum lipid levels, whereas the reduction in *Romboutsia* was also strongly associated with weight loss [61,62]. The fact that these two strains were observed as the main taxa in the Mod group and not detected in the BT and EFT groups also coincides with this. These microbial modifications at the genus level provide a plausible explanation for the amelioration of HFFCD-induced hyperlipidemia by BT and EFT.

Currently, the vast majority of research has concluded that a reduction in the abundance of *Akkermansia* in the gut is an important marker for a reduction in metabolic syndrome, and a recent review has referred to *Akkermansia muciniphila* as a next-generation probiotic [63,64]. Interestingly, our study demonstrated that *Akkermansia* abundance was significantly increased in hyperlipidemic mice, whereas it was maintained at a lower level in the Con, Pos, BT and EFT groups. Liu et al. also reported higher levels of *Akkermansia* in rats fed a high-fat diet [65]. These phenomena indicate that the effects of *Akkermansia* on organisms are not always positive. *Akkermansia* is mainly found in the intestinal mucosa and uses mucin as a carbon and nitrogen source, and mucin contributes positively to the protection of the intestinal barrier function [66]. Mice fed a fiber-free diet for a protracted period of time have been reported to cause intestinal inflammation, which is associated with *Akkermansia* phagocytosis of intestinal mucin [67]. Since chronic inflammation induces hyperlipidemia [58], amelioration of hyperlipidemia by supplementation with BT and EFT to reduce *Akkermansia* abundance may be mediated by alleviating the level of inflammation. Another explanation for the increase in *Akkermansia* in the Mod group may be that its presence decreases important enzymes that prevent hyperglycemia and hyperlipidemia [68]. Also, this could be another potential mechanism by which BT and EFT alleviate hyperlipidemia. In summary, current results conclude that *Akkermansia* is positively associated with hyperlipidemia and that BT and EFT interventions decreased *Akkermansia* abundance and alleviated hyperlipidemia. However, the role of *Akkermansia* in metabolic syndromes such as hyperlipidemia, obesity, and type 2 diabetes should require further investigation.

## 5. Conclusions

In conclusion, we indicate that microbial fermentation not only improves the flavor of BT but also enhances the hypolipidemic activity. This is evidenced by the fact that BT reduced serum lipid and liver lipid levels and that EFT enhanced these effects compared to BT. Mechanistic investigations have indicated that BT and EFT promote bile acid cycling, increase gene and protein expression of FXR, CYP7A1, and CYP27A1 in the liver, and promote ileal reabsorption of bile acids, enhancing ileal FXR and ASBT expression. In addition, BT and EFT remodel the gut microbiota. The alleviating effect of BT and EFT on hyperlipidemia may be associated with a decrease in the F/B ratio, a decrease in the abundance of Firmicutes, *Faecalibaculum* and *Romboutsia* and an increase in the abundance of Bacteroidetes. In particular, one of the mechanisms by which BT ameliorates dyslipidemia may be an increase in the abundance of cholesterol-lowering *Lactobacillus johnsonii.* And increased abundance of short-chain fatty acid-producing *Mucispirillum* and anti-inflammatory *Colidextribacter* are potential reasons for the hypolipidemic effect of EFT. In summary, we conclude that BT possesses favorable hypolipidemic efficacy and microbial fermentation which not only improves the flavor of black tea, but also strengthens the hypolipidemic activity of black tea. Our research offers recommendations for scientifically choosing teas and healthy tea consumption for hyperlipidemic individuals, as well as a theoretical underpinning for microbial fermentation to strengthen the hypolipidemic activity of tea and a novel idea for the development of natural tea products to alleviate hyperlipidemia. However, the hypolipidemic active components of tea and the role of *Akkermansia* in hyperlipidemia require further investigation.

## Data Availability

The original contributions presented in the study are included in the article/Supplementary Material, further inquiries can be directed to the corresponding author.

## Supplementary Materials

The following supporting information can be downloaded at: https://www.mdpi.com/article/10.3390/nu16070998/s1, Figure S1: Rarefaction curve of each group; Table S1: The compositions and energy densities of the diets; Table S2: Sequences of primers for RT-qPCR; Table S3: The sequence quality of 16S rRNA sequencing.

## Abbreviation

HFFCD: High-cholesterol diet; AMPK, Adenosine 5′-monophosphate-activated protein kinase; BT, Black tea; EFT, Microbial fermented black tea; TC, Total cholesterol; TG, Triglyceride; LDL-C, Low-density lipoprotein cholesterol; LDL-C, High-density lipoprotein cholesterol; TBA, total bile acids; ACE, Abundance-based coverage estimator; ASBT, Apical sodium-dependent bile acid transporter; FXR, Farnesoid X-Activated Receptor; CYP27A1, Cytochrome P450 27A1; CYP7A1, Cholesterol 7alpha-hydroxylase; GAPDH, Glyceraldehyde-3-phosphate dehydrogenase; HPLC, High-Performance Liquid Chromatography.
