# Supplementary figures and images for: Microbial Fermentation Enhances the Effect of Black Tea on Hyperlipidemia by Mediating Bile Acid Metabolism and Remodeling Intestinal Microbes
Source: Nutrients. 2024 Mar 28;16(7):998. doi: 10.3390/nu16070998 (PMC11013065; doi:10.3390/nu16070998)

**Figure S1.** Rarefaction curve of each group

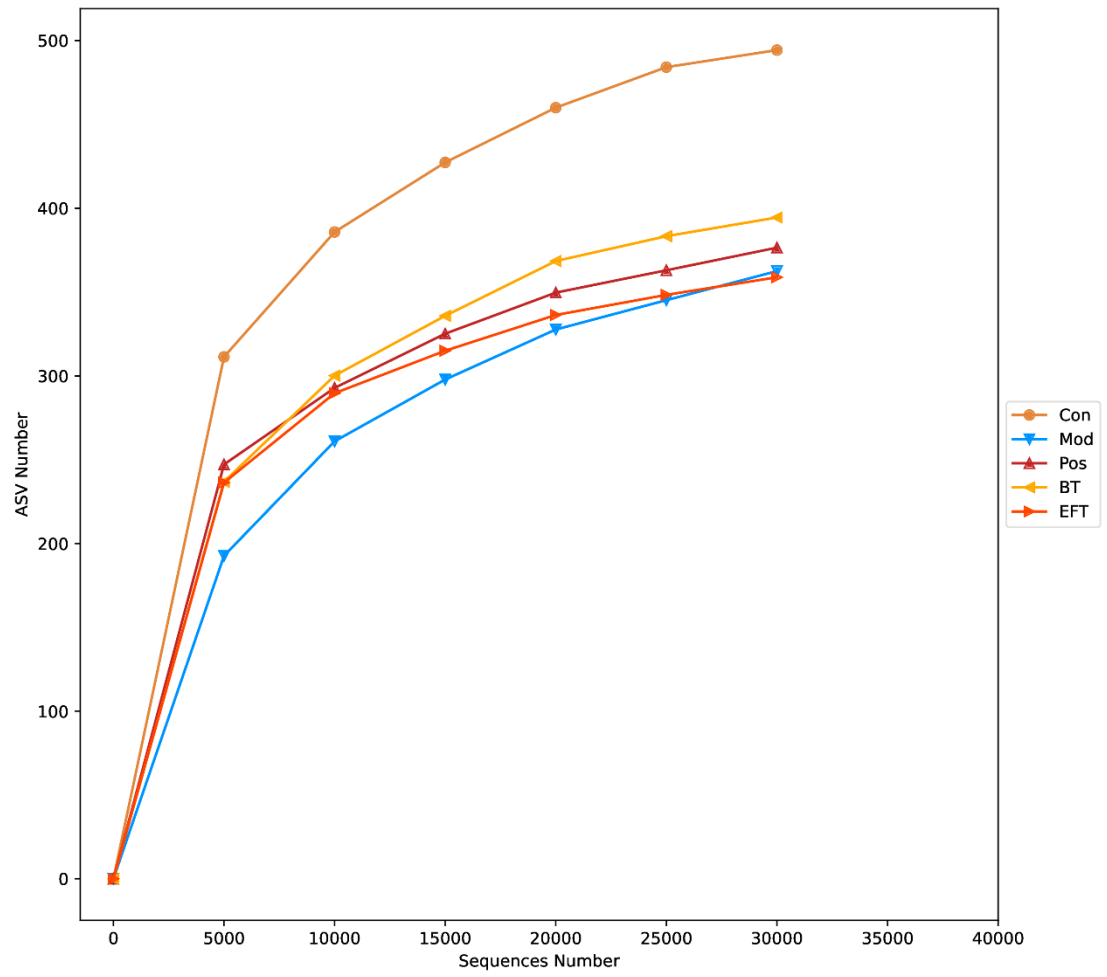

Supplement: Supplementary file 1 [file nutrients-16-00998-s001.zip › Supplementary Figure S1.pdf]
